# Gomisin N enhances TRAIL-induced apoptosis via reactive oxygen species-mediated up-regulation of death receptors 4 and 5

**DOI:** 10.3892/ijo.2011.1299

**Published:** 2011-12-15

**Authors:** HIROKI INOUE, PORNTHIP WAIWUT, IKUO SAIKI, YUTAKA SHIMADA, HIROAKI SAKURAI

**Affiliations:** 1 Division of Pathogenic Biochemistry, Institute of Natural Medicine, University of Toyama, 2630 Sugitani, Toyama 930-0194, Japan; 2 Department of Japanese Oriental Medicine, Graduate School of Medicine and Pharmaceutical Sciences, University of Toyama, 2630 Sugitani, Toyama 930-0194, Japan

**Keywords:** gomisin N, tumor necrosis factor-related apoptosis-inducing ligand, death receptor, reactive oxygen species

## Abstract

Pharmacological studies have revealed that lignans isolated from *Schisandra chinensis*, including gomisin N, show anticancer, anti-hepatotoxic, anti-oxidative and anti-inflammatory activities. Tumor necrosis factor-related apoptosis-inducing ligand (TRAIL) is an important member of the tumor necrosis factor superfamily with great potential in cancer therapy. The present study investigated whether pretreatment with gomisin N significantly enhanced TRAIL-induced cleavage of caspase-3, caspase-8 and PARP-1, which are key markers of apoptosis. Pretreatment with z-VAD-FMK, a pan-caspase inhibitor, was able to inhibit apoptosis enhanced by the combination of gomisin N and TRAIL. These results suggested that gomisin N could promote TRAIL-induced apoptosis through the caspase cascade. In search of the molecular mechanisms, we elucidated that such enhancement was achieved through transcriptional up-regulation of TRAIL receptors, death receptor 4 (DR4) and DR5. Neutralization of DR4 and DR5 could significantly reduce apoptosis induced by gomisin N and TRAIL. We also revealed that gomisin N increased the generation of reactive oxygen species (ROS). N-acetyl cysteine (NAC), an antioxidant, could inhibit ROS production and up-regulation of DR4 and DR5. Overall, our results indicated that gomisin N was able to potentiate TRAIL-induced apoptosis through ROS-mediated up-regulation of DR4 and DR5.

## Introduction

The fruit of *Schisandra chinensis* has been used traditionally to alleviate suffering from chronic cough and asthma and also to promote the production of body fluid to quench thirst and arrest sweating in East Asian countries. *S. chinensis* has also been employed in the treatment and prevention of some chronic diseases, such as inflammation, hepatitis and cancer. The major bioactive constituents of *S. chinensis* are lignans, including gomisins A, B, C, E, F, G, K, N and J, schisandrol B, and schisandrin C (1,2). Pharmacological studies revealed that lignans isolated from *S. chinensis* show anticancer, anti-hepatotoxic, anti-oxidative and anti-inflammatory activities (3–5). Gomisins A and N are dibenzo[a,c]cyclooctadiene lignans with R- and S-biphenyl configurations, respectively (6–8). Gomisin A shows anti-apoptotic activity and protects the liver from hepatotoxic chemicals (9). In contrast, gomisin N induces apoptosis of human hepatic carcinoma cells (10), and we have recently reported that gomisin N enhances TNF-α-induced apoptosis via inhibition of the NF-κB and EGFR survival pathways (11).

On the other hand, tumor necrosis factor (TNF)-related apoptosis-inducing ligand (TRAIL) is a member of the TNF superfamily that can initiate apoptosis via the activation of death receptor 4 (DR4) and DR5 (12,13). Since TRAIL induces apoptosis in transformed or tumor cells but not in normal cells, it is considered to be a promising cancer therapeutic agent, better than other TNF superfamily members, such as TNF and Fas ligand (14–17), which have no selectivity for normal and cancer cells. However, many types of cancer cells are resistant to TRAIL-induced apoptosis (18), therefore, it is important to overcome this resistance to expand the ability of TRAIL in cancer therapy. In this study, we focused on whether gomisin N was able to enhance TRAIL-induced apoptosis in HeLa cells and tried to explore the underlying molecular mechanisms.

## Materials and methods

### Antibodies and reagents

Anti-Bcl-xL, XIAP, Poly (ADP-ribose) polymerase-1 (PARP-1), caspase-8 and caspase-3 antibodies were purchased from Cell Signaling Technology, Inc. (Danvers, MA, USA). Antibodies against Bcl-2, caspase-9, cytochrome-c and β-Actin (C-11) were obtained from Santa Cruz Biotechnology, Inc. (Santa Cruz, CA, USA). Recombinant human TRAIL Apo II ligand was obtained from PeproTech, Inc. (Rocky Hill, NJ, USA). Gomisins A and N were purchased from Wako Pure Chemical Industries, Ltd. (Osaka, Japan). Annexin V was purchased from BioLegend, Inc. (San Diego, CA, USA). Anti-DR4 and anti-DR5 antibodies used for receptor blockage and z-VAD-FMK were obtained from Enzo Life Sciences, Inc. (Farmingdale, NY, USA).

### Cell culture and cytotoxicity assay

HeLa cells were maintained in Dulbecco’s modified Eagle’s medium (high glucose) supplemented with 10% fetal calf serum, 100 units/ml penicillin and 100 μg/ml streptomycin at 37°C in 5% CO_2_. Cell viability was quantified using the cell proliferation reagent WST-1 {4-[3-(4-iodophenyl)-2-(4-nitrophenyl)-2H-5-tetrazolio]-1,3-benzene disulfonate} (Dojindo, Kumamoto, Japan). HeLa cells were plated in 96-well microplates at 6×10^3^ cells/wells and then incubated for 24 h. Gomisin N-containing medium was added to the wells, and cells were incubated for 30 min and then stimulated with TRAIL. After 24-h incubation, 10 μl of WST-1 solution was added and absorbance was measured at 450 nm.

### Immunoblotting

Cells were treated with gomisin A, gomisin N and TRAIL, and whole-cell lysates were prepared with lysis buffer [25 mM HEPES pH 7.7, 0.3 M NaCl, 1.5 mM MgCl_2_, 0.2 mM EDTA, 10% Triton X-100, 20 mM β-glycerophosphate, 1 mM sodium orthovanadate, 1 mM phenylmethylsulfonyl fluoride (PMSF), 1 mM dithiothreitol (DTT), 10 μg/ml aprotinin and 10 μg/ml leupeptin]. Cell lysates were collected from the supernatant after centrifugation at 14,000 rpm for 10 min. Cell lysates were resolved by 10% sodium dodecyl sulfate-polyacrylamide gel electrophoresis and transferred to an Immobilon-P-nylon membrane (Millipore). The membrane was treated with Block Ace (Dainippon Pharmaceutical Co., Ltd., Suita, Japan) and probed with primary antibodies. The antibodies were detected using horseradish peroxidase-conjugated anti-rabbit, anti-mouse and anti-goat immunoglobulin G (Dako) and visualized with an enhanced chemiluminescence system (Amersham Biosciences). Some antibody reactions were carried out in Can Get Signal solution (Toyobo).

### Analyses of apoptotic cells by Annexin V-FITC

Cells pretreated with gomisin N (100 μM) for 30 min were treated with TRAIL (100 ng/ml) for 6 h. After harvesting, the cells were washed twice with 1,000 μl FACS buffer and resuspended in 500 μl FACS buffer containing 2.5 mM CaCl_2_ and 1 μg Annexin V-FITC for 15 min in the dark on ice. The samples were analyzed with the FACSCalibur System (BD Biosciences).

### Real-time RT-PCR

Total RNAs were prepared using the RNeasy Mini kit (Qiagen). First-strand cDNAs were synthesized by SuperScript II reverse transcriptase (Invitrogen, Carlsbad, CA, USA). The cDNAs were amplified quantitatively using SYBR Premix Ex Taq (Takara). The primer sequences are summarized in Table I (19). Real-time quantitative RT-PCR was performed using an ABI PRISM 7300 sequence detection system (Applied Biosystems). All data were normalized to GAPDH mRNA.

### Measurement of intracellular ROS

Reactive oxygen species (ROS) generation was measured by flow cytometry following staining with a chloromethyl derivative of dichloro dihydro-fluorescein diacetate (CMH_2_DCFDA; Invitrogen). Briefly, HeLa cells pretreated with gomisin N (100 μM) for 30 min were treated with TRAIL (100 ng/ml) for 2.5 h. The cells were stained with 5 μM CMH_2_DCFDA for 30 min at 37°C and harvested, and fluorescence intensity was analyzed using the FACSCalibur System (BD Biosciences).

### Statistical analysis

All values are presented as the mean ± SD and were analyzed by one-way analysis of variance (ANOVA) followed by the Tukey-Kramer test. P<0.05 was accepted as significant.

## Results

### Gomisin N augments TRAIL-induced apoptosis in HeLa cells

**Figure 1:**
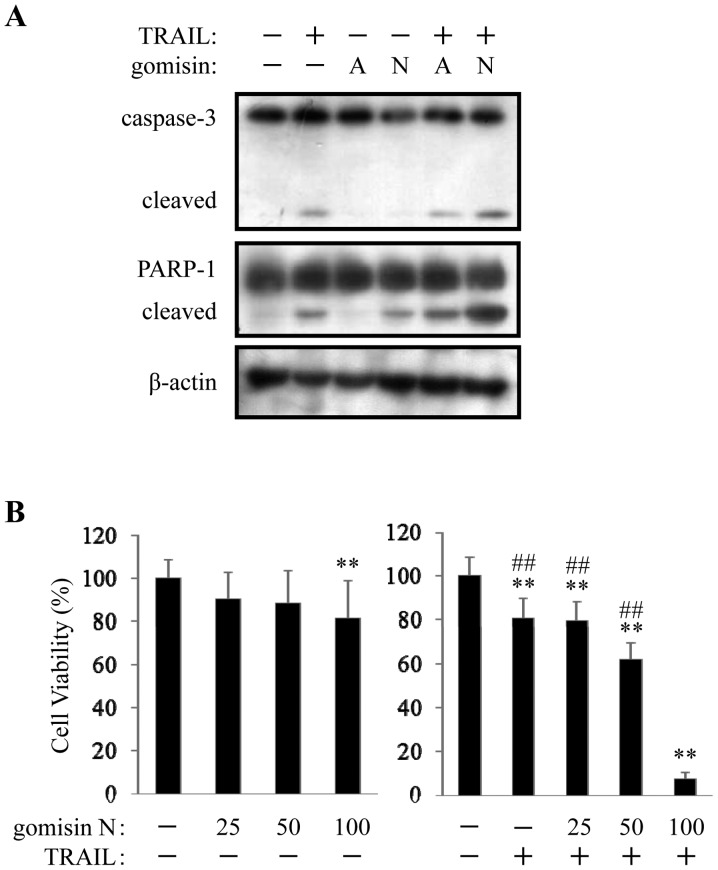
Effects of gomisins A and N on TRAIL-induced cell death. (A) HeLa cells were pretreated with 100 μM gomisin A or gomisin N for 30 min, followed by treatment with TRAIL (100 ng/ml) for 3 h. Cell lysates were collected and subjected to Western blot analysis to detect the cleavage of caspase-3 and PARP-1. (B) HeLa cells were treated with the indicated dosed of gomisin N for 30 min, followed by TRAIL treatment for 24 h. Cell viability was determined by WST-1 assay. Data are presented as the mean ± SD of twelve independent experiments; ^**^P<0.01 vs. control group; ^##^P<0.01 vs. combined treatment group with 100 μM gomisin N and TRAIL.

**Figure 2:**
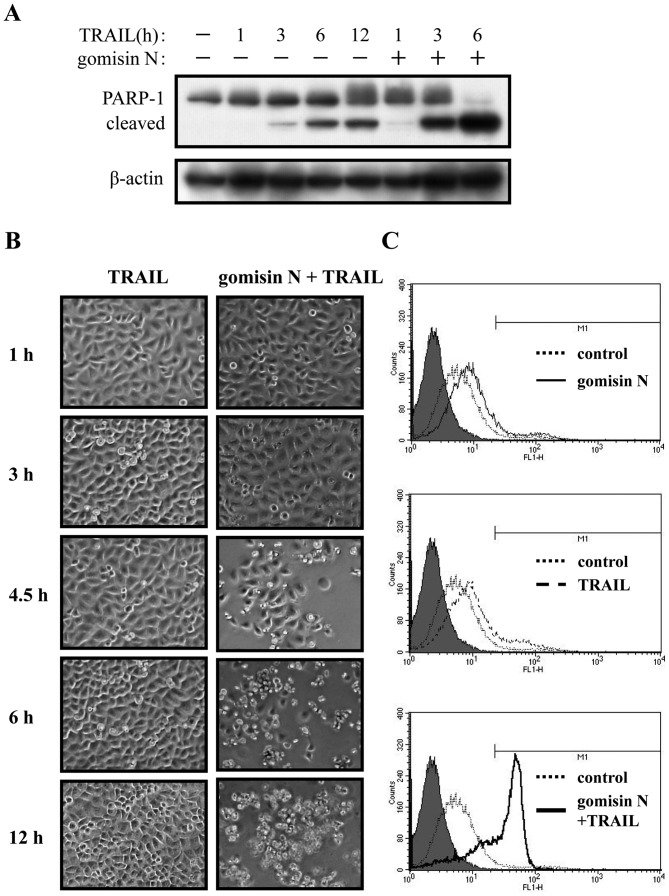
Pretreatment with gomisin N promotes TRAIL-mediated apoptosis. (A) Hela cells were pretreated with 100 μM gomisin N for 30 min and then treated with TRAIL (100 ng/ml) for the indicated hours. Cell lysates were collected and subjected to Western blot analysis to detect the cleavage of PARP-1. (B) Gomisin N morphologically enhanced the cell death induced by TRAIL in HeLa cells. Left, cells treated with TRAIL for the indicated hours; right, cells pretreated with gomisin N for 30 min, followed by TRAIL for the indicated hours. Representative images of cells were photographed using a normal light microscope. Magnification, ×100. (C) Cells were treated with gomisin N for 30 min, followed by TRAIL for 6 h. Cells were stained with Annexin V-FITC and analyzed by FACS.

Gomisins have been shown to induce apoptosis in cancer cells. We first confirmed the effects of gomisins A and N on TRAIL-induced apoptosis in HeLa cells. Gomisin A alone did not cause evident degradation of PARP-1, an important marker of apoptosis, and gomisin N and TRAIL alone induced cleavage of PARP-1 weakly, however, gomisin N, but not gomisin A, significantly enhanced TRAIL-induced cleavage of caspase-3 and PARP-1 (Fig. 1A). As shown in Fig. 1B, although gomisin N showed slight inhibition at a concentration of 100 μM, gomisin N enhanced TRAIL-induced cell death in a concentration-dependent manner. When treated with TRAIL alone at 100 ng/ml, cleavage of PARP-1 was detected in a time-dependent manner, and pretreatment with gomisin N accelerated TRAIL-induced cleavage of PARP-1 (Fig. 2A). Morphological changes were also observed after treatment with gomisin N and TRAIL together (Fig. 2B). The flow cytogram of Annexin V analysis is shown in Fig. 2C. For the control group, the percentage of apoptotic cells was 4.4%. After treatment with TRAIL or gomisin N alone, the percentage of Annexin V-positive cells was 14.7 or 10.1%, respectively, however, after co-treatment with gomisin N and TRAIL, the percentage of apoptotic cells markedly increased to 66.1%. These results indicated that gomisin N enhanced TRAIL-induced apoptosis.

### TRAIL-induced apoptosis is promoted by gomisin N through the caspase cascade

**Figure 3:**
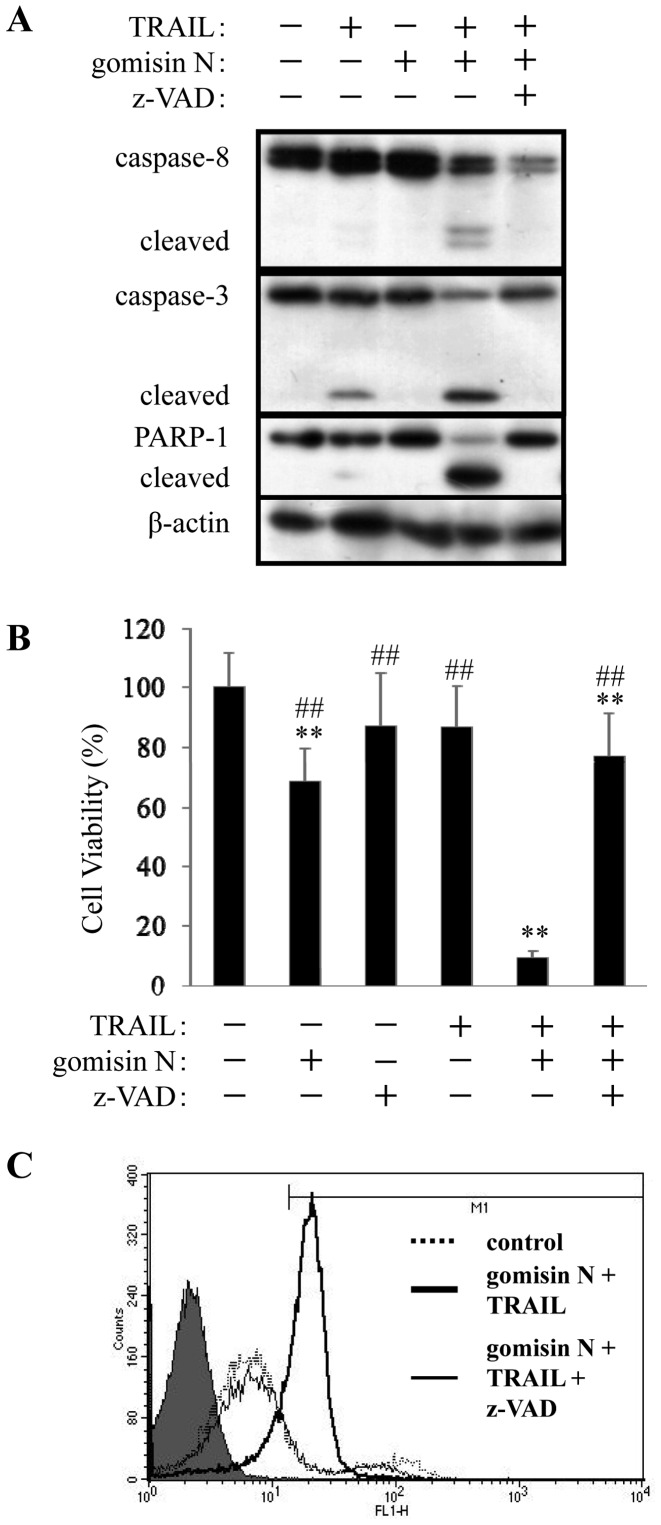
Combined treatment with gomisin N and TRAIL induces typical caspase-dependent apoptosis. (A) HeLa cells were pretreated with 10 μM z-VAD-FMK (z-VAD) for 30 min, followed by combined treatment with gomisin N (100 μM) for 30 min and then TRAIL (100 ng/ml) for another 3 h. Cells were collected to detect the cleavage of caspase-3, caspase-8 and PARP-1 by Western blot analysis. (B) HeLa cells were pretreated with z-VAD-FMK and gomisin N in a similar way and then treated with TRAIL for another 24 h. Cell viability was determined by WST-1 assay. Data are presented as the mean ± SD of six independent experiments; ^**^P<0.01 vs. control group; ^##^P<0.01 vs. combined treatment group with gomisin N and TRAIL. (C) HeLa cells were pretreated with z-VAD-FMK and gomisin N in a similar way and then treated with TRAIL for another 3 h. Cells were stained with Annexin V-FITC and analyzed by FACS.

Previous reports indicate that TRAIL-induced apoptosis is mainly executed through the extrinsic apoptosis pathway, involving caspase-8 and caspase-3 (20). We first examined the effect of gomisin N on TRAIL-induced activation of the caspase cascade. Fig. 3A shows that gomisin N alone had no effect on the degradation of caspase-8, caspase-3 and PARP-1, however, pretreatment with gomisin N significantly enhanced TRAIL-induced cleavage of caspase-8, caspase-3 and PARP-1. Moreover, pretreatment with z-VAD-FMK, a pan-caspase inhibitor, completely inhibited cleavage of caspase-8, caspase-3 and PARP-1 (Fig. 3A). In addition, WST-1 and Annexin V analyses showed that z-VAD-FMK also inhibited apoptosis induced by the combined treatment with gomisin N and TRAIL (Fig. 3B and C). These results suggested that gomisin N was able to promote TRAIL-induced apoptosis through the caspase cascade involving caspase-8 and caspase-3.

### Gomisin N enhances TRAIL-induced apoptosis via up-regulation of DR4 and DR5

**Figure 4:**
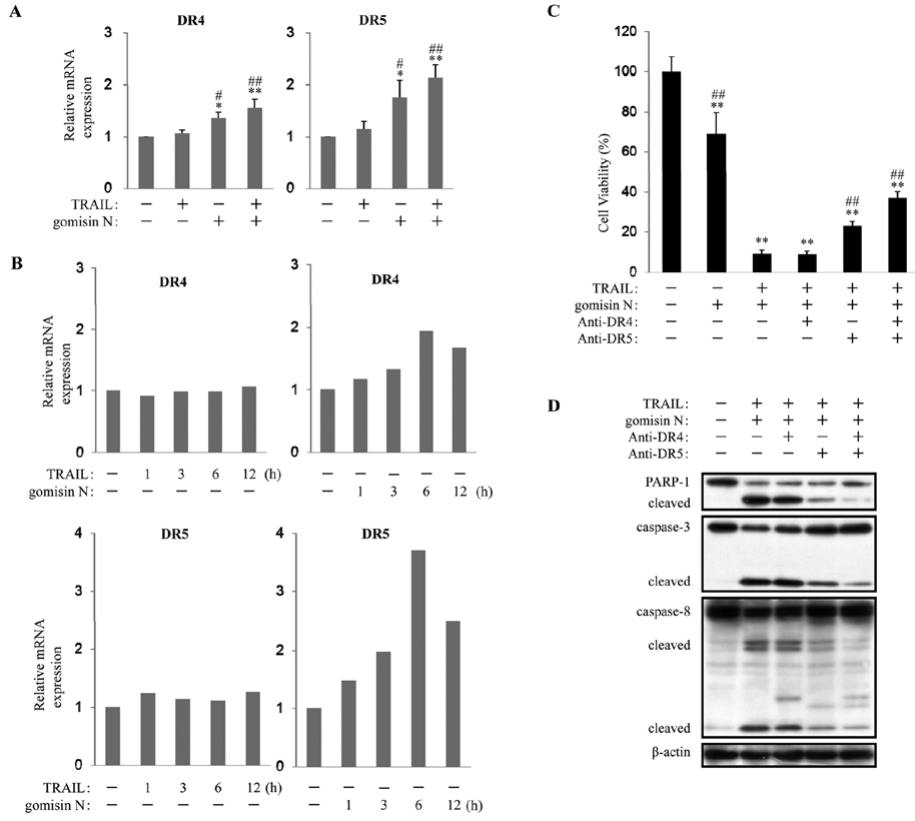
Gomisin N up-regulates mRNA expression of DR4 and DR5. (A) HeLa cells were pretreated with gomisin N (100 μM) for 30 min, followed by TRAIL (100 ng/ml) for 3 h. The mRNA levels of DR4 and DR5 were measured by real-time RT-PCR. Data are presented as the mean ± SD of three independent experiments; ^*^P<0.05, ^**^P<0.01 vs. control group, ^#^P<0.05, ^##^P<0.01 vs. treatment group with TRAIL. (B) HeLa cells were treated with TRAIL for the indicated hours (left). HeLa cells were pretreated with gomisin N for 30 min, followed by TRAIL for the indicated hours (right). The mRNA levels of DR4 and DR5 were measured by real-time RT-PCR. (C) HeLa cells were pretreated with DR4 and/or DR5 blocking antibodies (10 μg/ml) for 30 min, followed by combined treatment with gomisin N for 30 min and then TRAIL for another 24 h. Cell viability was determined by WST-1 assay. Data are presented as the mean ± SD of six independent experiments; ^**^P<0.01 vs. control group; ^##^P<0.01 vs. combined treatment group with gomisin N and TRAIL. (D) HeLa cells were pretreated with DR4 and/or DR5 blocking antibodies and gomisin N in a similar way and then treated with TRAIL for another 3 h. Cells were collected for detecting the cleavage of caspase-3, caspase-8 and PARP-1 by Western blotting.

It is well documented that decreased expression of TRAIL receptors DR4 and DR5 or increased expression of the decoy receptors DcR1 and DcR2 are responsible for TRAIL resistance in several cancer cell lines (13,20–22). To explore the underlying mechanism by which gomisin N enhanced TRAIL-induced apoptosis, we first detected the mRNA levels of DR4 and DR5 in HeLa cells treated with gomisin N. As shown in Fig. 4A, gomisin N significantly up-regulated the expression of DR4 and DR5 mRNA, and the combination of gomisin N and TRAIL accelerated the expression of DR4 and DR5. Furthermore, although TRAIL did not up-regulate mRNA levels of DR4 and DR5, gomisin N up-regulated DR4 and DR5 expression in a time-dependent manner until 6 h (Fig. 4B). Next to confirm the roles of DR4 and DR5 in TRAIL-induced apoptosis, TRAIL receptors were neutralized by anti-DR4 and DR5 blocking antibodies. As shown in Fig. 4C, WST-1 analysis demonstrated that DR4 blocking antibody was not able to inhibit TRAIL-induced cell death, however, after the neutralization of DR5, the percentage of cell viability increased to 23%. Moreover, after neutralizing both DR4 and DR5, the percentage increased to 37%. Western blot analysis showed that DR4 and DR5 blocking antibodies could inhibit the degradation of caspase-8, caspase-3 and PARP-1 similarly (Fig. 4D). These results suggested that gomisin N up-regulated DR4 and DR5 expression at the transcriptional level, and not only up-regulation of DR5 but also that of DR4 might be one of the mechanisms in the sensitization of TRAIL-induced apoptosis by gomisin N.

### Gomisin N up-regulates DR4 and DR5 expression by increasing ROS

**Figure 5:**
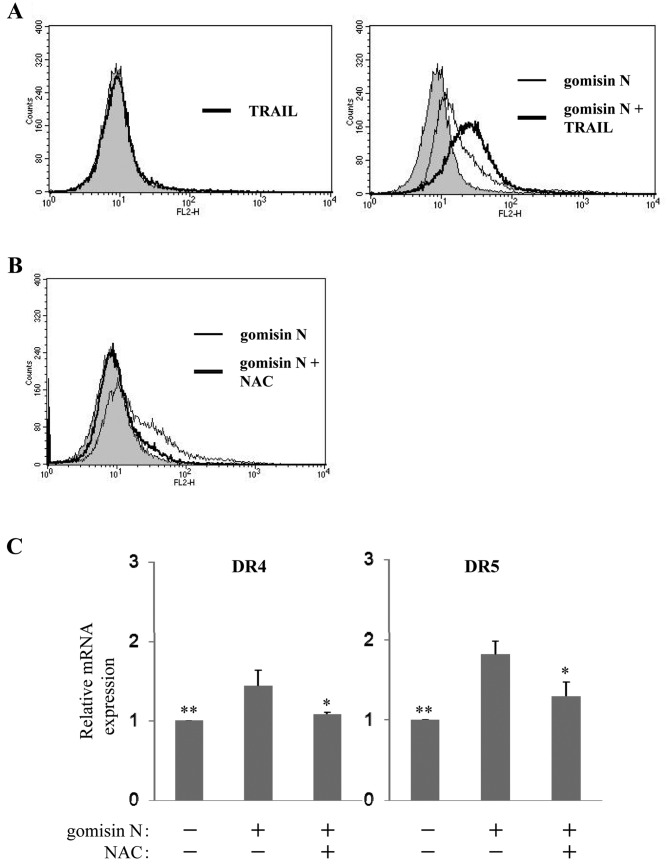
Gomisin N induces generation of ROS and up-regulation of DR4 and DR5 is dependent on ROS generation. (A) HeLa cells were pretreated with gomisin N (100 μM) for 30 min, followed by treatment with TRAIL (100 ng/ml) for 2.5 h. Cells were stained with 5 μM CMH_2_DCFDA for 30 min and analyzed by FACS. (B) HeLa cells were pretreated with 5 mM NAC for 30 min, followed by treatment with gomisin N for 3 h. Cells were stained with 5 μM CMH_2_DCFDA for 30 min and analyzed by FACS. (C) HeLa cells were treated with NAC (5 mM) followed by treatment with gomisin N for 3.5 h. The mRNA levels of DR4 and DR5 were measured by real-time RT-PCR. Data are presented as the mean ± SD of three independent experiments; ^*^P<0.05, ^**^P<0.01 vs. treatment group with gomisin N.

It has been reported that several natural products have ROS-generating activity and ROS are related to the up-regulation of TRAIL receptors (23–25). Here, we measured intracellular ROS levels treated with gomisin N. As shown in Fig. 5A, after treatment with TRAIL alone, the intracellular ROS level was almost the same as that of control cells without stimulation; however, after treatment with gomisin N, the ROS level increased significantly and co-treatment with gomisin N and TRAIL accelerated ROS production. Moreover, pretreatment with N-acetyl cysteine (NAC), an antioxidant, markedly reduced ROS production (Fig. 5B) and also significantly inhibited up-regulation of DR4 and DR5 induced by gomisin N (Fig. 5C). These findings indicated that up-regulation of DR4 and DR5 induced by gomisin N was dependent on ROS generation.

### Gomisin N does not markedly affect the mRNA expression of TRAIL decoy receptors, and protein expression of the intrinsic apoptosis pathway

**Figure 6:**
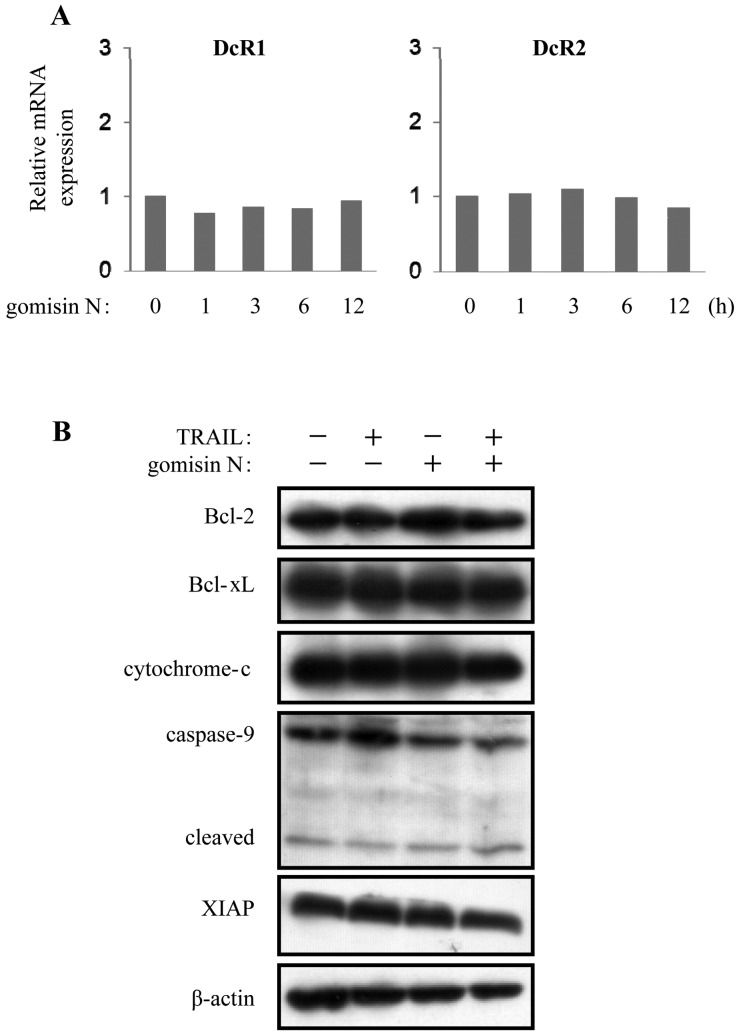
Effect of gomisin N on TRAIL decoy receptors and various apoptosis regulatory proteins. (A) HeLa cells were treated with gomisin N (100 μM) for the indicated hours. The mRNA levels of DcR1 and DcR2 were measured by real-time RT-PCR. (B) HeLa cells were pretreated with gomisin N for 30 min, followed by treatment with TRAIL (100 ng/ml) for 3 h. Cell lysates were collected and subjected to Western blot analysis to detect apoptosis regulatory proteins.

To investigate whether TRAIL decoy receptors were relevant to the sensitization of TRAIL-induced apoptosis by gomisin N, we examined mRNA expression of DcR1 and DcR2. As shown in Fig. 6A, gomisin N did not change the expression of DcR1 and DcR2. We also examined the protein levels of the intrinsic apoptosis pathway. Bcl-2, Bcl-xL, XIAP, cytochrome c and caspase-9 were not significantly changed by gomisin N treatment in HeLa cells (Fig. 6B). These findings suggested that gomisin N did not affect the mRNA expression of TRAIL decoy receptors and the intrinsic apoptosis pathway.

## Discussion

The effectiveness of chemotherapeutic drugs in cancer treatment has been limited by systemic toxicity and drug resistance. The distinct ability of triggering apoptosis in many types of human cancer cells while sparing normal cells makes TRAIL an attractive agent for cancer therapy, however, resistance to TRAIL-mediated apoptosis in cancer cells is a limitation in its clinical application as a cancer therapeutic agent. Thus, researchers are currently seeking TRAIL sensitizers to overcome resistance to TRAIL in cancer cells (18). A number of chemical compounds, including some natural products, have been identified as effective sensitizing agents to TRAIL-induced apoptosis (23,26–36). Gomisin N, a dibenzocyclooctadiene lignan isolated from the fruit of *S. chinensis*, has been reported as an anticancer drug candidate. Recent study demonstrated that gomisin N inhibited proliferation and induced apoptosis in human hepatic carcinomas (10). We have shown that gomisin N could sensitize TNF-α-induced apoptosis in HeLa cells (11), and the findings from this study provided substantial evidence that gomisin N was also capable of sensitizing TRAIL-induced apoptosis in HeLa cells. Thus, this study presents a novel anticancer effect of gomisin N and enhances the possibility of TRAIL in clinical application.

In the present study, to explore TRAIL sensitivity in human cervical cancer cells, HeLa cells were treated with 100 ng/ml TRAIL for 24 h. As shown in Fig. 1B, the viability of HeLa cells treated with TRAIL alone was 81%, but when treated with gomisin N (100 μM) and TRAIL, the percentage of viability decreased significantly to 7%. Analyses of apoptotic cells by Annexin V-FITC are shown in Fig. 2C. It was revealed that cells induced to undergo apoptosis by TRAIL alone were only 14.7%, but after treatment with gomisin N and TRAIL, the percentage of apoptotic cells increased to 66.1%. These results suggested that HeLa cells were resistant to TRAIL-induced apoptosis and gomisin N could promote TRAIL-induced apoptosis. To clarify the signaling pathway of apoptosis induced by TRAIL, we characterized the caspase-dependent pathway. The activation of caspase-8, caspase-3 and PARP-1 confirmed that the cell death induced by gomisin N and TRAIL was caspase-dependent apoptosis. As shown in Fig. 3, the pan-caspase inhibitor (z-VAD-FMK) was able to prevent PARP-1 cleavage and apoptosis induced by combined treatment with gomisin N and TRAIL, therefore, it was clarified that the apoptosis induced by gomisin N and TRAIL was caspase-dependent. Gomisin A enhanced the cleavage of PARP-1 induced by TRAIL slightly, but did not augment the cleavage of caspase-3 induced by TRAIL (Fig. 1A), therefore it was necessary to investigate the molecular mechanisms of gomisin A in enhancing the cleavage of PARP-1.

The expression level of death receptors (DR4 or DR5) plays a key role in determining the cell fate in response to TRAIL (20–22). There are numerous reports that the up-regulation of DR4 or DR5 could sensitize TRAIL-resistant cells to TRAIL-induced cell death (37,38). In this study, we showed for the first time that gomisin N increased DR4 and DR5 expression in HeLa cells (Fig. 4A and B). When we neutralized only DR4 by using a blocking antibody, the percentage of cell viability was not recovered, as compared with that of the combination of gomisin N and TRAIL. However, pretreatment with both DR4 and DR5 blocking antibodies inhibited the cell death induced by gomisin N and TRAIL more strongly than that of only DR5 (Fig. 4C), so we believed that both DR4 and DR5 up-regulated by gomisin N played key roles in sensitizing HeLa cells to TRAIL-induced apoptosis.

ROS generation has been proposed to be involved in the up-regulation of TRAIL receptors (23–25). The present study revealed that the mechanism by which gomisin N induced up-regulation of DR4 and DR5 was through the production of ROS. The antioxidant (NAC) could abolish the up-regulation of TRAIL receptors by gomisin N (Fig. 5C), therefore ROS production led to the up-regulation of DR4 and DR5, caspase cascade and eventually enhanced cell death.

In summary, we showed that gomisin N overcame TRAIL resistance through ROS-mediated up-regulation of DR4 and DR5 expression. Gomisin N might be useful to increase TRAIL efficacy in the treatment of malignant tumors.

## Figures and Tables

**Table I:** Sequences of RT-PCR primers.

Genes	Forward	Reverse
GAPDH	GCACCGTCAAGGTGAGAAC	ATGGTGGTGAAGACGCCAGT
DR4	ACAGCAATGGGAACATAG	GTCACTCCAGGGCGTACAAT
DR5	GCACCACGACCAGAAA	CACCGACCTTGACCAT
DcR1	GTTTGTTTGAAAGACTTCACTGTG	GCAGGCGTTTCTGTCTGTGGGAAC
DcR2	CTTCAGGAAACCAGAGCTTCCCTC	TTCTCCCGTTTGCTTATCACACGC
